# Oombarl Oombarl Joorrinygor—Slowly Slowly Moving Forward: Reflections From a Cross‐Cultural Team Working Together on the See, Treat, Prevent (SToP) Trial in the Kimberley Region of Western Australia

**DOI:** 10.1002/hpja.70025

**Published:** 2025-03-03

**Authors:** Tracy McRae, Janella Isaac, Hannah Thomas, Stephanie Enkel, Abbey Ford, John Jacky, Slade Sibosado, Kelli McIntosh, Marianne Mullane, Alexandra Whelan, Rebecca Dalton, Juli Coffin, Jonathan Carapetis, Roz Walker, Asha C Bowen

**Affiliations:** ^1^ School of Medicine University of Western Australia Perth Western Australia Australia; ^2^ Wesfarmers Centre of Vaccines and Infectious Diseases The Kids Research Institute Australia Nedlands Western Australia Australia; ^3^ Ardyaloon Community Kimberley Western Australia Australia; ^4^ The Kids Research Institute Australia Nedlands Western Australia Australia; ^5^ Ngangk Yira Institute for Change Murdoch University Perth Western Australia Australia; ^6^ Department of Infectious Diseases Perth Children's Hospital Nedlands Western Australia Australia; ^7^ Menzies School of Health Research Charles Darwin University Darwin Northwest Territories Australia; ^8^ University of Notre Dame Fremantle Western Australia Australia

**Keywords:** aboriginal health, children's health, health promotion, qualitative research, skin infections

## Abstract

**Introduction:**

Reflexivity is crucial for researchers and health professionals working within Aboriginal health. Reflexivity provides a tool for non‐Aboriginal researchers to contribute to the broader intention of reframing historical academic positivist paradigms into Indigenous research methodologies (IRM) to privilege Aboriginal voices in knowledge construction and decision‐making. This practice requires researchers to transition from safe and familiar research environments into unfamiliar and uncomfortable spaces. This uncomfortable space is often referred to as the ‘third space’—the ‘in‐between’ space that can be turbulent and difficult to navigate. However, it is also a productive space where new collaborations are created, and ideas can emerge. This manuscript provides reflections from a cross‐cultural team working on a transdisciplinary healthy skin program—the See, Treat, Prevent (SToP) Trial in Aboriginal communities in the Kimberley region of Western Australia (WA). Cultural mentors guided our team to work in an Oombarl Oombarl (steady steady) way to navigate the cultural interface between familiar biomedical elements and unknown health promotion activities. Our third space was the intangible space in‐between the S, T and P of the SToP Trial.

**Methods:**

Narratives were collected through semi‐structured interviews and yarning sessions. All participants provided written consent for audio recording; in one instance, consent was provided to record graphically. A thematic analysis aligning with the question guide was conducted.

**Findings:**

Reflections include team members' experiences of learning the Oombarl Oombarl way, individually and collectively. Initially, most team members revealed it was challenging to work in an Oombarl Oombarl way, having to move out of the safe, familiar research environment into the unknown community‐led health promotion space. This in‐between space became our third space—the uncomfortable space where we relinquished ‘control’ of research agendas and learnt to work to the rhythm of Aboriginal communities in WA's Kimberley region.

**Conclusion:**

Reflexivity is necessary when working in a cross‐cultural context. In Aboriginal homeland communities situated in remote settings, researchers benefit from being ‘on the ground’ to enable trust and genuine relationships to be developed. Visits on Country provide a rich experiential learning experience and a space for story sharing and yarning. Cultural guidance and two‐way learning partnerships with cultural mentors assist non‐Aboriginal researchers in understanding and adhering to cultural protocols and community processes. Allowing sufficient time to build relationships and flexible timelines are important considerations when developing research grants and protocols.

**So What?:**

Our findings demonstrate the importance of building genuine relationships and yarning on Country with Aboriginal communities to build health promotion knowledge together. Making meaning of health literacy can only evolve through two‐way learning partnerships where Aboriginal people guide the process. Our research reveals a novel approach to developing meaningful health promotion initiatives and resources on Country that centralise local Aboriginal language, artwork and community context.

## 1 Introduction

The juxtaposition of objectivity and subjectivity often creates challenges for researchers [1]. Researchers' perspectives and worldviews frequently influence how data is collected, interpreted, and represented, which has often resulted in criticism towards academic rigour and the role of reflexivity in the research process. However, reflexivity is a crucial tool for researchers and health professionals working within Indigenous health [2, 3]. Reflexivity provides a mechanism for non‐Indigenous researchers to contribute to the broader intention of reframing historical academic positivist paradigms into IRM [4, 5, 6] to privilege Indigenous voices [2]. In an Australian Aboriginal^1^ context, challenging Western paradigms in research calls for non‐Aboriginal researchers to commit to understanding, learning and centralising Aboriginal worldviews within research methodologies [1, 3, 7]. The role of reflexivity is an ongoing critical reflection to understand where and how our knowledge has been constructed and shaped our worldviews and, in turn, influenced our research approaches [2]. A commitment to practising reflexivity also provides a space where we, as researchers, can examine the assumed power and privilege afforded to us due to positionality in our professions [7].

## 2 Positionality and Assumptions of Power

Historically, the concept of positionality in research assumed a researcher was either an ‘insider’ or ‘outsider’; each status possesses advantages and disadvantages. However, this perspective has changed recently. The once clearly defined boundaries between insider and outsider status have become progressively unclear [8]. Both insider and outsider positions are influenced by age, ethnicity, language, gender, social status and environment, reflecting our evolving lives, previous experiences and unique interpretations. Positionality is fluid, depending on situations and circumstances [8, 9]. In these instances, it can also be important for researchers to recognise that power is negotiated rather than assumed:

There is a growing body of literature around issues of positionality, power and knowledge construction and representation in qualitative research. However, as all researchers have discovered, there is no substitute for actual fieldwork where these issues are personally encountered in sometimes unanticipated, and oftentimes subtle ways … During fieldwork the researcher's power is negotiated, not given. (8 p. 406 and 409)

Negotiating the challenges of researcher positionality and power relations within cross‐cultural research environments involves self‐awareness, authentic engagement and respectful acknowledgement of diverse perspectives, priorities and expectations. Navigating this journey requires researchers to embrace feelings of uncertainty and vulnerability [3, 7]. Acknowledging these feelings is important in the learning process and requires researchers to step out of their comfort zones and embrace uncomfortable spaces [1, 3, 7, 10].

## 3 Embracing Uncomfortable Spaces

### 3.1 Numbers or Narratives

The debate between quantitative and qualitative research methods is not new. The quantitative approach to science requires numbers and statistical measurements to support a hypothesis and provide a causal explanation. The quantitative process is, however, an important learning journey for researchers in academia [11]. In this instance, researchers remain detached and impartial, a space that may be more ‘familiar’ or ‘safe’ for medical scientists compared to the unknowns of qualitative inquiry [1]. However, even in the scientific world of numbers, qualitative research methods can provide a deeper understanding of varying perspectives at the cultural interface, and assist with improving relationships and communication in healthcare settings [12].

Qualitative research, by nature, involves researchers immersing themselves in situations and relationships that are complex and unpredictable [13]. In an Aboriginal context, non‐Aboriginal scientists and researchers may feel more comfortable navigating Western research environments rather than delving into the ‘unknowns’ [1] of Aboriginal methods of inquiry out of fear of doing or saying the ‘wrong’ thing [14, 15]. However, to make fundamental changes through IRM to centralise Aboriginal worldviews and improve health outcomes, researchers need to apply empathetic listening and understanding of Aboriginal people's historical and contemporary realities [16, 17]. This process may be confronting for non‐Aboriginal researchers. Accepting the uncomfortable and hard ‘truths’ about the historical, political and social inequities Aboriginal people have endured might be even more challenging [17]. As Wright et al. [17] pointed out: ‘there is nowhere to hide in these spaces’.(p. 117) However, learning the ‘truths’ of Aboriginal people is not just about hearing the injustices; it is also about learning the strengths, humour, and resilience of Aboriginal people. Wright et al. [17] highlighted that ‘hearing stories of strength, humour, and self‐determination also challenges the stereotypes of us as victims, helpless or broken, needing to be fixed’.(p. 117) Researchers' willingness to immerse themselves [18] in uncomfortable spaces can bring new perspectives to old stereotypes. These new perspectives are fundamental for systemic change [17].

### 3.2 Yarning and Story Sharing for Aboriginal Truth‐Telling

Trust and relationships are foundational for working authentically [3] with Aboriginal communities. These are developed over time and involve travelling to homeland communities, being on Country and yarning [18] with Elders and community members. Yarning is a well‐recognised research method of inquiry for building relationships with Aboriginal people. It has been theorised as:

An informal and relaxed discussion through which both the researcher and participant journey together visiting places and topics of interest relevant to the research study. Yarning is a process that requires the researcher to develop and build a relationship that is accountable to Aboriginal people participating in the research … To have a yarn is not a one‐way process but a dialogical process that is reciprocal and mutual. (17 p. 38)

Genuinely participating in yarning is important for Aboriginal truth‐telling [18, 19, 20]. It requires researchers to ‘reposition’ themselves from a place of ‘assumed’ power to a place where power is negotiated [8]. Demonstrating vulnerability will be valuable to this learning process [1, 3, 10]. On Country, Aboriginal people are the knowers of the ‘truth’ [17, 19], and researchers require reflexibility, humility and the willingness to learn new truths and realities [1, 3]. For Aboriginal people, Country is culture and family and is itself, an identity and place where Aboriginal and non‐Aboriginal people can come together [21, 22]. Being invited on Country is a privilege [20] that is not just a cultural experience, but also a historical and political learning experience, one of resistance [17]. Centralising ‘Country’ within research methodologies is an important element of deconstructing historical colonial research practices and provides a unique learning experience [20]. The important aspect of taking the time to learn the truths and realities of Aboriginal communities is relinquishing control of research agendas [2] and trusting the guidance of Aboriginal leaders in the learning process. This learning process will undoubtedly create frustration for researchers. Wilson [3] noted:

If you go in with a set agenda then you will just get frustrated if you don't cover it, which often seems to happen, because things are more important to discuss. And because you get hung up on this, you don't appreciate the fantastic spontaneity that can occur if you just let the conversation wander.(p. 223)

Western positivist research paradigms that have privileged objectivity, rigidity and distance do not work with Aboriginal communities [10]. There must be some balance between the numbers and the narratives. Reflexivity is crucial for researchers to discover a balance between objective and subjective truths [3, 7, 10]. This discovery will likely create initial struggles; however, these efforts are needed for fundamental systematic change [16]. As Dudgeon and Fielder reflected (15 p. 407), ‘Staying in our “own corners” may be safe but leaves little scope for learning and changes nothing’.

### 3.3 Working in the ‘Third Space’

The notion of the ‘third space’ is not new [16, 23, 24]. Homi Bhabha characterises it as the turbulent in‐between spaces where everything happens:

The third space, in essence, is the fissure in between ostensibly seamless and stable places. It is a space that can be opened up, but the impulse to pin down, close or paste over is strong. There is no pure, homogeneous cultural place for Bhabha. Everything happens in between. (15 p. 400)

The third space is not tangible; it is the ‘in‐between’ spaces where new signs of identity, innovative collaborations between cultures and contestations emerge [23]. Dudgeon and Fielder [16] argue that ‘it is vital to struggle to open and celebrate third spaces in our everyday lives’.(p. 405) For the Yolngu people in the Northern Territory, *‘*Ganma’ is the metaphor used for the meeting place in‐between the salt and fresh waters where two cultures converge and work together [25]:

Ganma is the name of a lagoon where salt and fresh water meet. Water is a symbol of knowledge in Yolngu philosophy, and the metaphor of the meeting of two bodies of water is a way of talking about the knowledge systems of two cultures working together. Dr Raymattja Marika (1959–2008) (29 para 1)

Similarly, Haynes et al. [23] discovered that ‘weaving a mat we can all sit on’ was a productive third space to strengthen knowledge about rheumatic heart disease at the cultural interface [26].

For this research, the cultural interface was a healthy skin clinical trial aimed at reducing the burden of skin infections using a culturally responsive, transdisciplinary approach incorporating both treatment and prevention measures. The See, Treat, Prevent (SToP) Trial was conducted in Aboriginal communities in the Kimberley region of Western Australia. The aim of this manuscript is to contribute to the literature regarding the importance of reflexivity when working in a cross‐cultural environment. Reported here are the experiences from Aboriginal and non‐Aboriginal team members navigating the third space between the biomedical and prevention components of the SToP Trial. Importantly this manuscript describes a constructivist two‐way learning [27] journey where Aboriginal mentors have guided non‐Aboriginal researchers to work in a culturally responsive way to the rhythm of communities while adhering to the parameters of a clinical trial.

### 3.4 The SToP Trial

The SToP Trial was a collaboration between the Telethon Kids Institute, the Kimberley Aboriginal Medical Services, the WA Country Health Services–Kimberley and Nirrumbuk Environmental Health and Services [28]. The SToP Trial was the first healthy skin clinical trial aimed at reducing the burden of skin infections using a culturally responsive, transdisciplinary approach incorporating both treatment and prevention measures (Figure 2).

During the initial extensive consultations from 2014 to 2016, Kimberley‐based stakeholders, partners and communities involved in the trial articulated the high priority for health promotion and environmental health activities to complement the biomedical (See and Treat) components of the SToP Trial. The SToP Trial protocol describing the study design and primary outcomes is reported elsewhere [28]. The stepped‐wedge design was considered the most appropriate approach to ensure all communities received the intervention (Treat component) but at different time points. The community participatory action research [29] approach of the SToP Trial ensured that Aboriginal people governed the collection, ownership and application of data about their lands, communities and resources. The highest committee overseeing all aspects of the SToP Trial was the Partnership Steering Group, in which the Aboriginal members had a veto over decisions and shared decision‐making responsibility for the entire study.

### 3.5 A Cross‐Cultural Team

The SToP team comprised researchers who consistently conducted remote fieldwork and worked in two‐way partnerships with Aboriginal researchers from the Kulunga Aboriginal Unit (Kulunga).^2^ The dynamics of the fieldwork team varied depending on circumstances, including which community we were visiting, the time of year we were travelling and external factors that often impacted travel.^3^ Collectively, four team members identified as Aboriginal and seven identified as non‐Aboriginal (Kartiya).^4^ Kulunga team members were either born in the Kimberley and lived there with strong connections or were born elsewhere and living in the region at the time of the study. The SToP team had two members born elsewhere living in the Kimberley. The remaining five team members lived in Perth and travelled to and from the Kimberley throughout the study period.^5^ Female team members outnumbered males by 9:2. Professionally, the team's disciplines included biomedicine, education, social science and the resources sector. Collectively, we self‐identified as insiders or outsiders, or both depending on circumstances [9]. The challenge often lay in bridging the distinct worlds of working in fast‐paced, structured, clinical research to the contrasting rhythm of local Aboriginal community life with a more grounded way of knowing, doing and being [6]. This in‐between space created the third space we navigated the Oombarl Oombarl way.

### 3.6 Learning the Oombarl Oombarl Way in the Third Space

Bardi people we've always been about, “Don't rush,” it's about not rushing and thinking before you take the next steps. The old people one day used that term Oombarl Oombarl, and I first heard it when I was a child, it was teaching me to survive when we would go out on country, to be careful where I'm treading, look before I step, sort of thing, or just slow down, don't rush. So, they would use that word when I was a child, just teaching me what that was, and to growing up understanding what Oombarl Oombarl is about…If you get [Oombaarl Oombaarl], and if you're going to take it out now into the wider world and put it out there, you'll see that people will come back to you and say, ‘Oh yeah, well that's like this, and this, and this’. (Cultural Mentor)

**FIGURE 1:**
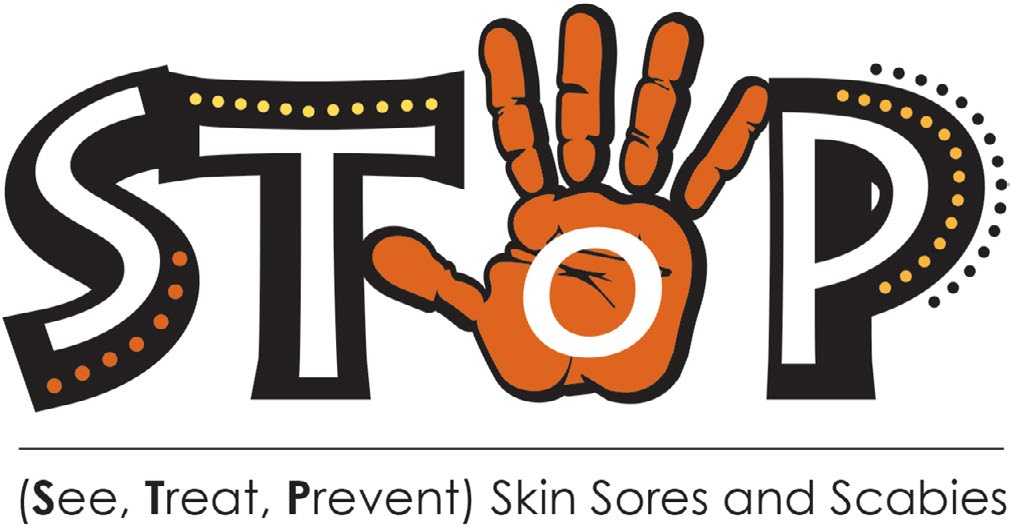
The see, treat, prevent (SToP) trial.

Figure 1 graphically represents a conversation between the first author and a cultural mentor, who was influential in guiding the team to navigate the often‐turbulent space in‐between the constructs of a clinical trial and the fluidity of community life. The third space was a journey of adaptation, understanding and continuous inquiry for coexistence as we often found ourselves outside our comfort zones, challenging our fears [7] and learning to relinquish our ‘assumed position of control’ that was often taken for granted due to our professional positionality as researchers. Identifying as a Pākehā^6^ woman born in New Zealand and embarking on cross‐cultural research, I acknowledge my worldviews and privileges that I brought to this research. This work is embedded within my qualitative PhD research project.

## 4 Methods

### 4.1 Participants and Data Collection

Underpinned by the philosophy of constructivism [30] to include multiple worldviews, narratives for this collective reflection were collated from the first author's reflective journal, reflective semi‐structured interviews [31] and yarning sessions [18] with SToP Trial and Kulunga team members. All participants were assured of confidentiality and advised that participation was voluntary. The first author conducted six semi‐structured interviews face‐to‐face in Perth, two interviews virtually via Microsoft Teams, and a yarning session in Broome and an Aboriginal community. The first author participated in a face‐to‐face semi‐structured interview in Perth conducted by a SToP Trial researcher. These semi‐structured interviews and yarning sessions allowed the authors to share reflections and experiences of learning the Oombarl Oombarl way in our research.

**FIGURE 2:**
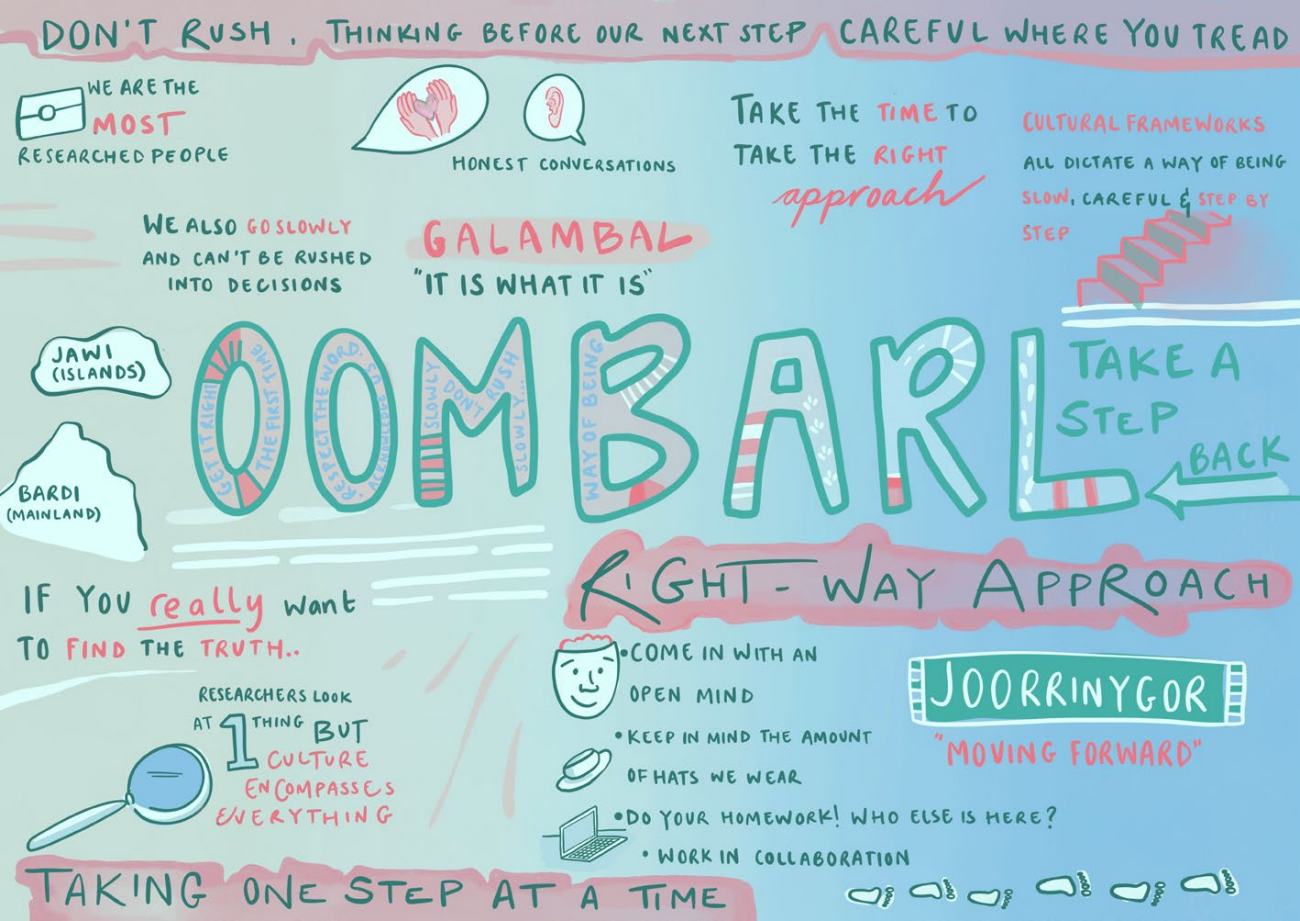
Graphic recording of the Oombarl Oombarl way developed by Neda Loh from Tuna Blue.

All interviews and yarnings were audio recorded with consent and saved as digital recordings in a de‐identified format. The audio recordings were transcribed verbatim and uploaded with the written responses into QSR NVivo (Version 12). One yarning session was graphically recorded, as shown in Figure 2 Each transcript was assigned a code number to protect participants' privacy. Transcripts and written responses were coded independently following the question guide, and specific theme codes were added when new themes emerged from the data. Transcripts were provided to participants for member checking and trustworthiness [32].

### 4.2 Ethics

This Project Was Approved by the Health Ethics Review Committees at the Child and Adolescent Health Service (Approval Number RGS0000000584), the Western Australian Aboriginal Health Ethics Committee (Reference Number: 819), University of Western Australia (Reference RA/4/20/4123), Catholic Education Western Australia (Reference Number: RP2017/57) and Department of Education (Reference Number: D18/0281633).

### 4.3 Findings

My reflections along with my cultural mentors' perspectives on Oombarl Oombarl are accompanied by those of nine participants who consented to share their experiences of working as part of the SToP Trial. In order to achieve the objectives of the study, questions focused on three topics: learning the Oombarl Oombarl way, working between the biomedical and prevention elements and, two‐way learning partnerships.

### 4.4 Learning the Oombarl Oombarl Way

When participants were asked what Oombarl Oombarl meant and how this concept of working influenced their journeys, some team members were familiar and comfortable with navigating this approach. However, the majority (six) participants articulated that learning the Oombarl Oombarl way required introspection to understand the differences between professional and personal identities. This introspection required a critical lens and willingness to let go of expectations and accept ‘what is’ rather than what ‘should be’.

#### 4.4.1 Personal Reflection

My first experience working in a ‘steady steady’ way was during a field trip in 2019 while travelling with a Kulunga team member and discussing the ‘agenda’ for visiting the community.

I was saying, ‘Right, you know, I need to talk to this many people, and we need to do this, and we have to get the BBQ set up, and we have to be done by this time’. And [name] was just like, ‘You've got to slow down, Sister, this is not about you’. And that set the scene for understanding the process of slowing down.

#### 4.4.2 Team Reflections

It's kind of interesting because I feel like [Oombarl Oombarl] comes naturally to me as an individual, like socially and so as a human in my kind of outside life, but it doesn't come naturally to me as a researcher, or it's getting better. But I think that's like a nature versus nurture thing and, this upbringing we have in academia is. (Participant 3)

Was it fast enough? No. Was it engaged enough? No. Was it immensely frustrating? Absolutely. Will it make a difference? I really hope so. And I think that value of making a difference is probably so core to the way I operate that I try my hardest to listen when being triggered, or when, you know, getting asked to pivot, or when pushback happens—or anything like that, and go, ‘Well, what can we learn here? How can we implement to the best of our ability?’ And I don't know if I ever will land at this, but trying to be kind to myself, and others, that ‘We're just learners. Science is about learning’. (Participant 4)

This concept of Oombarl Oombarl over time is very interesting to me, I guess it means a lot of things to me … It's a reminder to us as researchers that wait, stop, slow down. Like, remember why you're doing this. Remember what's important. (Participant 5)

### 4.5 We Found Ourselves Positioned ‘Outside Our Comfort Zones’

Regardless of insider or outsider positionality, ‘outside our comfort zones’ was where we often found ourselves. During the reflection interviews, participants described the different situations that were confronting and challenging for them. The diversity across the team meant we were all pushing the boundaries of our comfort zones, yet none of us articulated our feelings of discomfort at the time.

#### 4.5.1 Personal Reflection

I was incredibly anxious on starting this journey. I was very careful and guarded when yarning with the Elders. I was scared of ‘doing the wrong thing’.

We've had conversations in the past, I feel like if you go in, and I went in with a place of fear, of doing the wrong thing, of saying the wrong thing, of offending somebody, [but then] as it was pointed out to me, if we don't actually put ourselves out there, then we may never have those relationships built.

#### 4.5.2 Team Reflections

I do think in this whole process that transparency is pretty important because I think you learned their hard lessons and sometimes they're uncomfortable truths. (Participant 1)

And just letting things go and getting on with it. But I also think we're a very unique team in that we've seen each other at our best and our worst I feel. Like, some days in community where things haven't gone well, and you're sharing a room, you're in each other's space all the time. And then, you just crack a little bit but then I think we have been good at recognising that was a moment and just sort of letting it go. (Participant 2)

I would second guess every interaction; I would second guess every word that I said because I was very aware that I'm an outsider and I didn't want to affect or offend people. And I'm not naturally an outgoing, confident person and then having to be that constantly on trips is just exhausting, and being around people all the time is exhausting. And so, for people like us, there were just so many points where we're out of our comfort zone, and that's exhausting to deal with. (Participant 6)

It was a little bit—not—I'm not going to say ‘challenging’, but it was just new for me to work with a [different gender] all the time. It was a bit of a challenge at the start. It's—even just dealing with the different emotions and everything that go with it. (Participant 9)

### 4.6 The See Component of SToP Was More Comfortable and Familiar

Bridging the gap between the biomedical and social science disciplines was more challenging for biomedical researchers trained in quantitative data collection rather than social science methods of narrative inquiry. For biomedical researchers, working in the school environment was more secure and familiar than the ‘unknowns’ of the community‐led Aboriginal health promotion.

#### 4.6.1 Personal Reflection

And I think, what I saw with the team who were so comfortable working in the schools—because you got to school at a certain time, you knew when your breaks were, you knew where the kids were going to be, everything was there. But when you go to community and you spend the day, you don't know when you're going to eat, you don't know where you're going to sit to chat, you're not going to know who's [in community] to talk to. And I think even just that in itself, but also being and seeing things that you wouldn't necessarily see in an urban setting [can be challenging], you know?

#### 4.6.2 Team Reflections

And I think in many ways, those school surveillances were easier. And looking back as to why that might be, it's in a school environment, which is Western. It was very quantitative in nature. You could very much document. I think the surveillance side of it was so much easier because it was the comfortable zone. I can say that that was my comfortable zone, too. (Participant 1)

I feel more comfortable when I have more control. So, I think the See and Treat stuff came a bit more naturally and easily. And yeah, more difficult with the Prevent, just letting it be guided and having faith that it would work out. (Participant 2)

I guess in terms of when we're in community, it really hard to shift your expectation of what you're going to achieve in a day, and then get to the end of the day and not actually have anything quantitative that you can go back and report on. And it took a lot for me to shift from being mostly a surveillance team member to being more in the prevent space, and not beating myself up when nothing happened. (Participant 6)

### 4.7 Learning Flexibility—It May Not Look Like You Expected

The hybrid design of the SToP Trial required a structured approach to align with clinical trial protocols as well as allowing the community‐led health promotion to organically evolve. At times this approach was difficult, and participants shared reflections on shifting expectations and realigning research agendas to the rhythm of the communities.

#### 4.7.1 Cultural Mentors' Perspective

Okay, let's take the time to just relax, be more flexible, because if we restrict ourselves, we won't be able to move forward, we'll still keep coming back, coming back, coming back, without going forward. It's okay to take three steps forward and two steps back sometimes, but continuously doing that, it has a big impact. (Cultural Mentor)

#### 4.7.2 Personal Reflection

Consistently listening to communities and receiving guidance from cultural mentors highlighted the need for flexibility in our research approach. Being flexible was sometimes difficult, particularly when clinical trials by design are rigid for rigour and efficacy. It was difficult initially when I visited communities and took the time required to build relationships. In a Western research world, this could be (or is at times) perceived as doing ‘nothing’. That was a challenge for me, not being able to ‘quantify’ what I was doing when I measured my productivity against Western standards of research and protocols.

I think the very first few years, and we had COVID as well, which was, you know, we can't not appreciate that was a big part, but I think you live in this journey of feeling like you've not done anything. And that in the research world is not, you know, often well‐received when people are going, ‘Oh, what have you been doing?’ you know. [And you say], ‘Well, I went and had a yarn, you know, I didn't get any formal yarning done, but I sat on, you know, and I talked to these people, and I did that’. And then, in hindsight, when you look back, you know that you were doing things, but they just weren't in the tangible way that we report.

#### 4.7.3 Team Reflections

You have to go in and go. I'm just gonna sit with community. I'm just gonna listen. And if on the day all we have had a yarn about is maybe some of the beautiful artwork they're creating, that's okay like it's maybe not ticking boxes of what we went to achieve. (Participant 1)

And it was very, very, like thought out, not thought out, sorry, very strict around what you can and can't do. And to me, when I was observing, I could see, like, that you probably weren't getting the richest of data that you could get, because it was so time restricted as well. (Participant 9)

[I] just learned that you really if you wanna get the most out of, if you want to gain that community voice you can't put time limit on it. (Participant 11)

### 4.8 The Role of Cultural Guidance

Collaborating with Kulunga was a significant factor in building relationships in the community and following protocols, particularly in earlier fieldwork trips^7^ where Kulunga team members provided a cultural lens and mentoring to the SToP team. A two‐way partnership facilitated mutual trust and understanding of our unique worldviews and positionality in the study.

#### 4.8.1 Cultural Mentor Perspective

I'd say, obviously, Aboriginal people are the most researched people, and we are to a certain point researched out, I think. But my advice, not just for research, with anything really, try to look at things from a cultural perspective as best you can, and just remember the way we see things is we're constantly wearing our cultural lens. (Cultural Mentor)

#### 4.8.2 Personal Reflection

I've had the three different perspectives of travelling with [name], [name] and [name], and they all have been very different, but I've learnt something from them all. I've spent a lot of time in—in the car with [name] on my own, so I felt like even that in itself is a relationship—you know, when you start driving. And the same with [name] and [name] and I ended up travelling where we'd go for hours with not even saying anything because we'd talked for eight hours, you know, on the way out to [community name]. So, each have brought a different experience. I think in hindsight each have come in at the right time for the journey that I needed, because [name] gave me the confidence, [name] was more laid‐back. And then [name] was good at getting processes, you know, done with like, the books and stuff. And even though there was some challenging times, I look back now and think well, there was so much that I learned from just working with her as well.

#### 4.8.3 Team Reflections

And so, in terms of Kulunga and two‐way learning and things like that, I think it definitely needs to go both ways. I think there's a lot of things that I learnt working with the Kulunga team, and I think there's a lot of upskill, like equally. We've both been, on that kind of journey together, I guess, both learning. (Participant 5)

That's right. You're there to learn at the end of the day. And them mob wanna teach. Just like us people, when—us as Aboriginal people going into the white, or Western [way]. And, like, going down to the Institute for myself, I was—that scared the [redacted] out of me the first time, you know? (Participant 9)

### 4.9 Learning by Working in the Third Space

Individually and collectively, we shared the journey of finding a steadier, more considered approach to our work. It was a prolonged process, and we did not start that way. Through reflexivity and acknowledging our worldviews, pushing our comfort zones and having a willingness to work in a new way that challenged what we learn in academia, we evolved into the third space of Oombarl Oombarl.

#### 4.9.1 Personal Reflection

For me, understanding the rhythms of daily life in a community made the Oombarl Oombarl way less challenging. As a team, we observed each other develop confidence and trust in Aboriginal people guiding the process and a willingness to relinquish our ‘assumed control’ and let go of our expectations and accept what is rather than what ‘should be’

Understanding the rhythms of community is really important for the way that you can work in an [Oombarl Oombarl] way. I think that's been my biggest learning, is that you just don't apply it as a thing, it's kind of just a way of being?

Even when I've worked with [name], you know, her and I are very similar in so many respects, and in other ways we're not, but we complement each other. And just the growth that I saw in [name] from the first time that she had to work in the community setting as opposed to the safety [familiarity] of the school, I'll say, was just incredible to [see that]—I know that [name] still, and we all still try not to take it personally, and it's hard not to, but the more that you just understand the rhythms of community and understand the [Oombarl Oombarl] way, it becomes less challenging.

#### 4.9.2 Team Reflections

I've really learnt to lean into, ‘it will probably not go to plan, something will probably go wrong’. Something will probably go absolutely right in a way I never expected it to, and I think out of anything relationships grow from it, whether it's from peers or from relationships in communities you get stronger. (Participant 1)

I've noticed, like, a progression in myself and in the team with that. So, at first it was very, like I felt personally responsible if we didn't get an outcome that we wanted. And I took a lot onto myself. And therefore, tried really hard to make things happen … And yeah, like we'd do our summaries on the SToP chat and, you try and make it sound as good as you can because that's what people see. But I found with time I've let go of a lot of that and just accepted that things happen the way they happen. (Participant 2)

I think it's been like marked a shift, not just in, our research practice, but, in our team culture I think a little bit in the way that we engage with each other and stuff. And again, we don't always get it exactly right, but I feel like even just the way we hold meetings now is more conversational and, taking it as it flows. We still have our sort of little list of agenda items there, but very regularly we don't follow them and everyone's sort of okay with that more. And I think that we've just kind of come as a team to this, like, collective understanding of what that looks like for us without necessarily specifically articulating it ever. (Participant 3)

We learnt so much, and it was really heartwarming to see people so willing to share their culture with us. And I think we're really lucky because the communities that we visit are so different and there's so much uniqueness about them. So, I think that trip, it just allowed me to slow down and—and see every moment for what it was. (Participant 6)

### 4.10 Oombarl Oombarl Joorrinygor—Slowly Slowly Moving Forward

When asked what advice participants would provide for other teams, the strategies included self‐care, cultural awareness training and respecting each other's unique worldviews. Flexibility to restructure agendas and time frames within protocols is important for developing trust and relationships with Aboriginal communities. Participants believed being on Country and engaging in emphatic listening and reciprocal story sharing is essential for culturally responsive health promotion activities for healthy skin.

### 4.11 Cultural Mentor's Perspective

It's about going forward, because when you're saying, creating it's something that you're doing, trying to achieve, it's an action. And like I said, there's no word for change, but there's this word that we use that's Joorrinygor, which means moving forward, future, whatever, going forward. So, what I'm thinking [Oombarl Oombarl] Joorrinygor is, ‘Slowly, slowly, going forward’. (Cultural Mentor)

### 4.12 Personal Reflection

I feel like if you really want to do the research in a way that brings in people's voices, you have to go and sit with people in the community. And everybody on the team should do that, including the CIs [chief investigators]. I feel like there needs to be a bit of a, I don't know the term, but where people actually make the effort to understand. I think it's very different for us on the ground. But you can't explain what it's like out in some of those communities. You know, like you can't come back and go, ‘Oh, you know, it's—it was really hard, it's hot, it's dusty’. People don't really appreciate it until they experience it themselves.

### 4.13 Team Reflections

I think you also need to have the right people on your team. I think that's tricky. I think a lot of people, even if their hearts in the right place, they may not really know what it's like to work in this context and that it's not going to be easy at times. And I think you've got to have people who are willing to adapt and be open to changing and open to feedback. I think that's important. (Participant 5)

So, I say this to most people, really, is that time—allow that time at the start and build those relationships. Which is, sort of, key to how I see getting rich data … and making it more meaningful, for yourselves and the people that you're working with, you know? And, you know, sometimes you can probably get stuck with, you know, the investigators stuck down in Perth, and they don't come up and see what's actually happening on the ground … they need to come up. It's a no—it's non‐negotiable. They need to see the people that they're trying to work with. They need to understand the context around everything that they're trying to do. And in different communities, there's different concepts. (Participant 9)

What looks like and what happens in a community is very different to what happens in an institution, and those contracts and how we needed to work through those cause … [In a] geographical location like out there in the [region name], the work that we did was very different, even from those three different communities and how we had to unpack, that and work with what we had like, you know; obviously we had to work with a contract [from organisation name]. (Participant 10)

To put it into your protocol that you have that flexibility to be able to make those changes from the start … and realise from the start that things are not gonna go to plan and then you can word it that your whenever you're entering community you're going to do whatever surveillance or whatever you're doing that you will be responsive to what the community asks you to do and follow their cultural protocols. (Participant 11)

## 5 Discussion

Our study reports reflections from a cross‐cultural team navigating the third space of working within a clinic trial in Aboriginal communities living remote from urban areas in the Kimberley region of WA. As a team, we did not start working in an Oombarl Oambarl way. Feelings of frustration [3] and fear [1] were experienced across the team at different times and for various reasons. Embracing these uncomfortable spaces was essential for challenging Western research paradigms and relinquishing the control often assumed by researchers in academia [3, 7]. In the field, positionality and power are not a given; they are negotiated [8]. Researchers must commit to the ongoing practice of reflexivity and critiquing our worldviews for a genuine shift in power dynamics [2, 3, 10]. This transition will increase researchers' feelings of vulnerability, but being willing to work through these feelings is fundamental for systematic change [15].

The SToP Trials' clinical design, timelines and protocols required following a structured process that did not always correspond with the rhythm of Aboriginal community life. The biomedical and Prevention components of SToP were conducted simultaneously throughout the study. However, the See and Treat components functioned within the parameters of the trial design, whereas the Prevention component evolved organically with the communities. While recognising numbers and narratives are equally important for healthy skin, this disjuncture of methodologies in achieving the aims of the SToP Trial created a sense of anxiety for the team. Working in the quantitative space was more familiar for many team members in contrast to gathering data through narratives [1], which resulted in learning the uncomfortable truths that were often confronting for the team [16]. There is space for both quantitative and qualitative methods in an Aboriginal context. Importantly, qualitative methods can provide a deeper understanding of perspectives to help improve communication and healthcare [26] However, researchers must commit to an ongoing cycle of critical reflection to transform old stereotypes with inherent biases into new perspectives.

Importantly, through continuous self‐reflection and guidance from cultural mentors, Elders and community members, the rhythm of communities became our rhythm. All team members shared feelings of unease when transitioning from the familiar spaces into the unknown, whether moving between biomedical and social science activities or working in Western environments. Ongoing self‐awareness and collective reflections throughout our research journey revealed the uncomfortable situations that dictated our positionality and power. Despite this, a willingness and commitment to spend time on Country and in these ‘in‐between’ spaces provided opportunities for the emergence of new collaborations and innovations [15, 16].

Time is a crucial element for building trust and relationships with Aboriginal communities [3]. Our findings support the literature regarding the need for flexible time frames and letting go of ‘what is’ rather than expecting ‘what should be’ [3, 10]. An inflexible approach does not work in Aboriginal communities. Future research teams working in the same context will require commitment and understanding of the critical need to centralise Aboriginal worldviews and work to the rhythm of communities in a genuinely respectful way. Challenging our fears and letting go of expectations evolved over time. Through a constructivist journey, we learned the Oombarl Oombarl way and its potential to create a productive third space. This transition occurred in the community context and the Perth research environment. Like Ganma, the meeting of two waters and ‘weaving a mat we can all sit on’, we learnt the Oombarl Oombarl way which signifies the ‘hand’ in‐between the See, Treat and Prevent in SToP.

## 6 Conclusion

There is no substitute for the firsthand experience of working with Aboriginal communities situated remote from services. For research to be conducted in a culturally appropriate and meaningful way, all team members, including chief investigators, must experience immersion on Country with communities. While challenging and confronting, it is essential to developing relationships and trust. From an Indigenist research perspective, allowing for flexibility, unrestricted timelines and the ability to work in rhythm with Aboriginal communities is non‐negotiable for future research programs. Receiving cultural guidance and working in two‐way partnerships with Aboriginal people helps to negotiate tense and uncomfortable spaces that will occur at the cultural interface. We share our unique experiences to assist future research teams conducting research at the cultural interface, aiming to achieve equitable health outcomes. Changing historical research paradigms necessitates all individuals transitioning from well‐known safe places into unknown and uncomfortable spaces to establish culturally responsive health research.

## Conflicts of Interest

The authors declare no conflicts of interest.

## Data Availability

Research data are not shared.

## Endnotes

^1^
The authors acknowledge Aboriginal and Torres Strait Islander people are the Indigenous peoples of Australia however in the context of this work we use the term Aboriginal people.

^2^
The Kulunga Aboriginal Unit's role is to be the primary professional support service that links Aboriginal communities with researchers working at the Telethon Kids Institute. Referred to as ‘Kulunga’ the unit has a team based in Broome overseeing research conducted in the remote Kimberley communities.

^3^
External factors included COVID‐19 travel restrictions, illness, cultural and personal factors.

^4^
Kartiya is a common term among Kimberley people to refer to non‐Aboriginal people.

^5^
While many people volunteered and worked on the project, seven core SToP team members and three Kulunga team members are central to this narrative.

^6^
Pākehā is a Māori‐language term for New Zealanders primarily of European descent.

^7^
2019, 2020, 2021.
